# Carboxyxanthones: Bioactive Agents and Molecular Scaffold for Synthesis of Analogues and Derivatives

**DOI:** 10.3390/molecules24010180

**Published:** 2019-01-05

**Authors:** João Ribeiro, Cláudia Veloso, Carla Fernandes, Maria Elizabeth Tiritan, Madalena M. M. Pinto

**Affiliations:** 1 Laboratório de Química Orgânica e Farmacêutica, Departamento de Ciências Químicas, Faculdade de Farmácia, Rua de Jorge Viterbo Ferreira, 228, 4050-313 Porto, Portugal; joaobigi@gmail.com (J.R.); claudiaazevedo7@gmail.com (C.V.); elizabeth.tiritan@iscsn.cespu.pt (M.E.T.); 2 Interdisciplinary Centre of Marine and Environmental Research (CIIMAR), Edifício do Terminal de Cruzeiros do Porto de Leixões, Av. General Norton de Matos s/n, 4050-208 Matosinhos, Portugal; 3 Cooperativa de Ensino Superior, Politécnico e Universitário (CESPU), Instituto de Investigação e Formação Avançada em Ciências e Tecnologias da Saúde (IINFACTS), Rua Central de Gandra, 1317, 4585-116 Gandra PRD, Portugal

**Keywords:** xanthone scaffold, carboxyxanthone derivatives, carboxyxanthone analogues, bioactivities

## Abstract

Xanthones represent a structurally diverse group of compounds with a broad range of biological and pharmacological activities, depending on the nature and position of various substituents in the dibenzo-γ-pyrone scaffold. Among the large number of natural and synthetic xanthone derivatives, carboxyxanthones are very interesting bioactive compounds as well as important chemical substrates for molecular modifications to obtain new derivatives. A remarkable example is 5,6-dimethylxanthone-4-acetic acid (DMXAA), a simple carboxyxanthone derivative, originally developed as an anti-tumor agent and the first of its class to enter phase III clinical trials. From DMXAA new bioactive analogues and derivatives were also described. In this review, a literature survey covering the report on carboxyxanthone derivatives is presented, emphasizing their biological activities as well as their application as suitable building blocks to obtain new bioactive derivatives. The data assembled in this review intends to highlight the therapeutic potential of carboxyxanthone derivatives and guide the design for new bioactive xanthone derivatives.

## 1. Introduction

**Figure 1:**
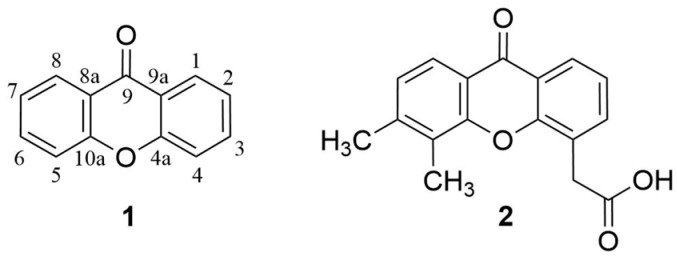
Xanthone scaffold and numbering (**1**) and DMXAA (**2**).

Xanthones (9*H*-xanthen-9-ones) are an important class of oxygenated three-membered heterocyclic compounds with a dibenzo-γ-pyrone scaffold (**1**, Figure 1) [1]. Over the years, considerable interest has been attracted in xanthone derivatives mainly because of their diverse range of biological/pharmacological activities [2,3,4,5]. The xanthone scaffold is considered a privileged structure [6,7], which can belong to the pharmacophoric moiety for the activity exhibited or as a substituent group associated with other chemical cores to modulate diverse biological responses [3].

Naturally-occurring xanthones can be found as secondary metabolites in diverse terrestrial sources including higher plants, fungi, lichens [8,9] as well as isolated from marine invertebrates, such as sponges, tunicates, mollusks and bryozoans, in addition to algae and marine microorganisms (cyanobacteria and fungi) [10,11]. They comprise a variety of different types of substituents in certain positions of the xanthone scaffold, leading to a vast diversity of biological/pharmacological activities [3] as well as different physicochemical and pharmacokinetic properties [12,13], being a remarkable basis for the discovery of new potential drug candidates.

Currently, there are many drugs on the market and in clinical trials, which were isolated or based on natural products [14,15,16], highlighting that natural compounds, such as xanthone derivatives, have always been a source of inspiration for the discovery of new therapeutic agents [14]. Some commercially available extracts with human health promotion properties present xanthone derivatives in composition [9]. Nevertheless, biosynthetic pathways only allow the presence of certain groups in specific positions on the xanthone scaffold. Therefore, the total synthesis strategy allows access to structures that otherwise could not be reached within the natural product as a launching platform for molecular modification [17]. In fact, with proper synthetic pathways, many other substituents can be introduced into the xanthone scaffold affording the development of more diverse compounds for biological activity and structure-activity relationship (SAR) studies [18], as well as other applications such as preparation of fluorescence probes [19,20] or stationary phases for liquid chromatography [21,22,23]. For the last several years, the isolation and synthesis of new bioactive xanthone derivatives using different synthetic methodologies has remained in the area of great interest of our group, as exemplified in [24,25,26,27,28,29,30,31,32,33,34,35].

Among the large number of natural and synthetic xanthone derivatives, those containing a carboxylic group have shown great significance in medicinal chemistry. A remarkable example is 5,6-dimethylxanthone-4-acetic acid (DMXAA, Vadimezan, ASA404, **2**, Figure 1), a simple carboxyxanthone derivative, which reached phase III clinical trials towards antitumor activity [36].

This review aims to describe the research findings on biological and pharmacological activities of natural and synthetic carboxyxanthone derivatives. Their applications as suitable chemical substrates to obtain new analogues and derivatives are also presented.

## 2. Natural Carboxyxanthone Derivatives

Typically, natural xanthones are classified in six main groups, depending on the nature of the substituents in the xanthone scaffold: simple xanthones, glycosylated xanthones, prenylated xanthones, *bis*-xanthones, xanthonolignoids and miscellaneous [3,9]. More recently, Masters and Bräse [8] subdivided the natural xanthones in monomers and dimers/heterodimers. Regarding the structural characteristics of natural carboxyxanthone derivatives, in this review they are classified into simple carboxyxanthone derivatives, prenylated carboxyxanthone derivatives, caged carboxyxanthone derivatives, and carboxyxanthone derivatives bound or fused to polysubstituted oxygenated heterocycles.

### 2.1. Simple Carboxyxanthone Derivatives

#### 2.1.1. 2-Hydroxy-6-Methyl-8-Methoxy-9-oxo-9*H*-Xanthene-1-Carboxylic Acid (**3**) and 2-Hydroxy-6-Hydroxymethyl-8-Methoxy-9-Oxo-9*H*-Xanthene-1-Carboxylic Acid (**4**)

**Figure 2:**
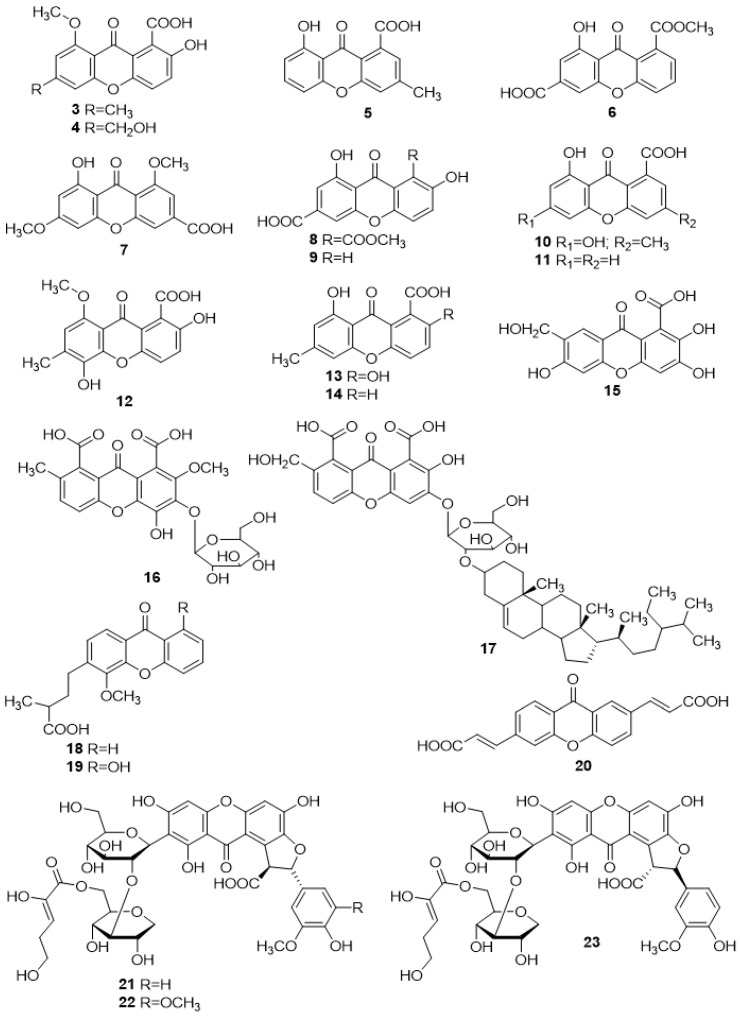
Structures of simple carboxyxanthone derivatives (**3**–**23**).

Healy et al. [37] described, in 2004, the isolation of two new carboxyxanthones, 2-hydroxy-6-methyl-8-methoxy-9-oxo-9*H*-xanthene-1-carboxylic acid (**3**) and 2-hydroxy-6-hydroxymethyl-8-methoxy-9-oxo-9*H*-xanthene-1-carboxylic acid (**4**) (Figure 2), from the strain *Xylaria sp*., of the tree *Glochidion ferdinandi*. These compounds were tested for toxicity in a brine shrimp (Artemia salina) lethality assay and for antimicrobial activity against *Escherichia coli*, *Streptococcus pneumonia*, *Enterococcus faecalis*, *Pseudomonas aeruginosa*, *Straphylococcus aureus* and *Candida albicans*, showing no activity in either of the assays [37]. In 2016, Beattie et al. [38] tested these compounds for antimicrobial activity against several organisms, including *Escherichia coli*, *Staphylococcus aureus*, *Candida albicans*, *Cryyptococcus neoformans* and *Cryptococcus gatti* as well as cytotoxicity against mammalian cells. Although compound **4** was inactive, compound **3** showed mild antifungal activity against *Cryptococcus neoformans* and *Cryptococcus gatti* [38].

#### 2.1.2. Monodictyxanthone (**5**)

In 2007, Krick et al. [39] isolated a new carboxyxanthone, monodictyxanthone (**5**) (Figure 2), from the fungus genus *Monodictys putredinis* and tested it in a series of bioassays for potential cancer chemopreventive activities. The results showed dose-dependent Cytochrome P450 1A activity inhibition and a slight inhibition of the enzyme aromatase [39].

#### 2.1.3. 8-(Methoxycarbonyl)-1-Hydroxy-9-Oxo-9*H*-Xanthene-3-Carboxylic Acid (**6**)

The carboxyxanthone 8-(methoxycarbonyl)-1-hydroxy-9-oxo-9*H*-xanthene-3-carboxylic acid (**6**) (Figure 2), isolated from a culture broth of the mangrove endophytic fungus *Penicillium sp.* from the bark of *Acanthus ilicifolius Linn*, by Shao et al. in 2008 [40], was tested for cytotoxicity against human epidermoid carcinoma and multidrug-resistant human epidermoid carcinoma of the nasopharynx; however, no activity in either assays was observed [40].

#### 2.1.4. Yicathin C (**7**)

Sun et al. [41] reported, in 2013, the isolation of yicathin C (**7**) (Figure 2), from the inner tissue of the marine red alga *Gymnogongrus flabelliformis*. Yicathin C (**7**) was assayed for antibacterial and antifungal activities using a standard agar diffusion test. Inhibitory activity against *E. coli*, *S. aureus* and *C. lagenarium* was observed [41]. In addition, it was found that this marine carboxyxanthone exhibited weak brine shrimp (Artemia salina) toxicity [41].

#### 2.1.5. 2,8-Dihydroxy-1-Methoxycarbonyl-9-Oxo-9*H*-Xanthene-6-Carboxylic Acid (**8**) and 2,8-Dihydroxy-9-Oxo-9*H*-Xanthene-6-Carboxylic acid (**9**)

The isolation of the carboxyxanthone 2,8-dihydroxy-1-methoxycarbonyl-9-oxo-9*H*-xanthene-6-carboxylic acid (**8**) (Figure 2) was firstly described, in 2014, from the marine derived fungus *Penicillium citrinum* SCSGAF 0167 strain [42]. This compound was tested as potential cathepsin B inhibitor; however, it showed no inhibitory activity [42]. In 2015, Ma et al. [43] reported the isolation of compound **8** from the fungal endophyte *Aspergillus versicolor*. Further biological activity evaluation showed a strong inhibitory activity against α-glucosidase enzyme [43]. Recently, Liao et al. [44] isolated the same compound (**8**) from an endophytic fungus *Arthrinium arundinis* of *Anoectochilus roxburghii* as well as a new carboxyxanthone, 2,8-dihydroxy-9-oxo-9*H*-xanthene-6-carboxylic acid (**9**) (Figure 2).

#### 2.1.6. 6,8-Dihydroxy-3-Methyl-9-Oxo-9*H*-Xanthene-1-Carboxylic Acid (**10**)

In 2010, Li et al. [45] reported the isolation of 6,8-dihydroxy-3-methyl-9-oxo-9*H*-xanthene-1-carboxylic acid (**10**) (Figure 2) from the toxigenic fungus *Penicillium oxalicum*. To the best of our knowledge, no activities were described for this compound.

#### 2.1.7. Globosuxanthone D (**11**)

Wijeratne et al. [46] isolated the carboxyxanthone globosuxanthone D (**11**), from the fungal strain *Chaetomium globosum* of the cactus, *Opuntia leptocaulis*, in 2006, and tested it against seven human solid tumor cell lines; however, no activity was observed (Figure 2).

#### 2.1.8. 2,5-Dihydroxy-8-Methoxy-6-Methyl-9-Oxo-9*H*-Xanthene-1-Carboxylic Acid (**12**)

The carboxyxanthone 2,5-dihydroxy-8-methoxy-6methyl-9-oxo-9*H*-xanthene-1-carboxylic acid (**12**) (Figure 2) was isolated by Davis et al. [47], in 2006, from the endophytic fungus *Xylaria sp. FRR 5657*; however, no biological activity was reported so far.

#### 2.1.9. Pinselic Acid (**13**)

Pinselic acid (**13**) (Figure 2) was firstly isolated, in 1953, by Munekata [48] from the fungal strain *Penicillum amarum*. In 2004, Healy et al. [37] isolated the same compound (**13**) from a microfungus of *Xylaria sp.* genus. To the best of our knowledge, no activity studies were performed for this compound.

#### 2.1.10. 8-Hydroxy-6-Methyl-9-Oxo-9*H*-Xanthene-1-Carboxylic Acid (**14**)

In 2014, Abdissa et al. [49], isolated the carboxyxanthone 8-hydroxy-6-methyl-9-oxo-9*H*-xanthene-1-carboxylic acid (**14**) (Figure 2), from the roots of *Bulbine frutescens*. Additionally, this compound (**14**) demonstrated to be inactive against KB-3-1 cervix carcinoma human cell line [49].

#### 2.1.11. 2,3,6-Trihydroxy-7-Hydroxymethylene Xanthone-1-Carboxylic Acid (**15**) and Glycosilated Analogues (**16**–**17**)

2,3,6-Trihydroxy-7-hydroxymethylene xanthone-1-carboxylic acid (**15**), 2-methoxy-4-hydroxy-7-methyl-3-*O*-β-d-glucopyranosyl xanthone-1,8-dicarboxylic acid (**16**), and 2-hydroxy-7-hydroxymethylene xanthone-1,8-dicarboxylic acid 3-*O*-β-d-glucopyranosyl(2′→3′′)-3′′-*O*-stigmast-5-ene (**17**) (Figure 2) were described in 2011 by Singh et al. [50] upon isolation from the seeds of *Rhus coriaria L.* All compounds were further tested for antifungal activity against *Aspergillus flavus*, *Candida albicans*, and *Penicillum citrinum* strains. Carboxyxanthones **16** and **17** were effective, showing inhibitory growth activity for all three fungal strains. The only exception was compound **15** which was ineffective against *Penicillum citrinum* [50].

#### 2.1.12. Scriblitifolic Acid (**18**) and Teysmannic Acid (**19**)

The isolation of scriblitifolic acid (**18**) (Figure 2), from the heartwood of *Calophyllztm scriblitifolium*, was first described by Jackson et al. [51], in 1967. Later, in 2000, Kijjoa et al. [52] reported the isolation of a new carboxyxanthone derivative, teysmannic acid (**19**), along with scriblitifolic acid (**18**), from the wood of *Calophyllum teysmmannii var. inophylloide* from Southern Thailand. To the best of our knowledge, no activities were described for both compounds.

#### 2.1.13. (2*E*,2′*E*)-3,3′-(9-Oxo-9H-Xanthene-2,6-Diyl)Diacrylic Acid (**20**)

(2*E*,2′*E*)-3,3′-(9-oxo-9*H*-xanthene-2,6-diyl)diacrylic acid (**20**) (Figure 2), was isolated from the leaves of *Santolina insularis*, in 2005, by Cottiglia et al. [53]. This carboxyxanthone was proven to have moderate anti-inflammatory activity against croton oil-induced ear oedema in rats, after topical application [53].

#### 2.1.14. Glomexanthones A–C (**21**–**23**)

The isolation of glomexanthones A–C (**21**–**23**) (Figure 2), from an ethanol extract of *Polygala glomerata*, was described by Li et al., in 2014 [54]. These compounds were subjected to neuroprotection bioassays in human neuroblastoma SK-N-SH cells and showed moderate neuroprotective effects on l-Glutamic acid-induced cellular damage [54].

### 2.2. Prenylated Carboxyxanthone Derivatives

#### 2.2.1. 2,8-Di-(3-Methylbut-2-Enyl)-1,3,8-Trihydroxy-4-Methyl-Xanthone (**24**)

**Figure 3:**
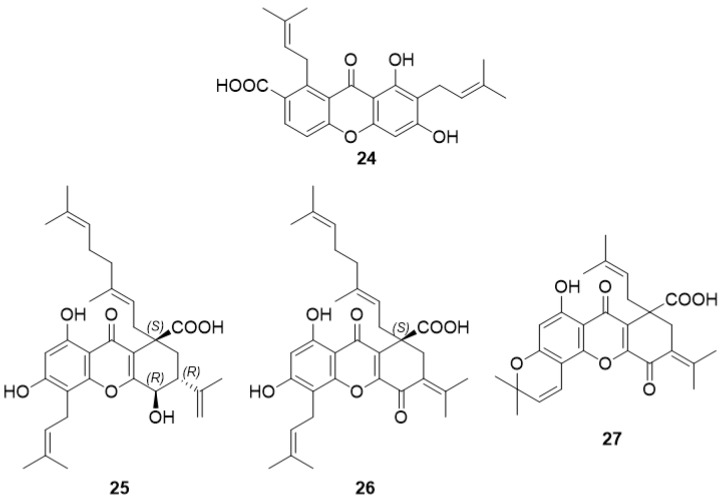
Structures of prenylated carboxyxanthone derivatives (**24**–**27**).

Gopalakrishnan and Balaganesan [55] reported, in 2000, the isolation of a new carboxyxanthone, 2,8-di-(3-methylbut-2-enyl)-1,3,8-trihydroxy-4-methyl-xanthone (**24**) (Figure 3), from the fruit hulls of *Garcinia mangostana*. To the best of our knowledge, no activity was reported for compound **24**.

#### 2.2.2. Oliganthic Acid A (**25**), Oliganthic Acid B (**26**), and (±)-Oliganthic Acid C (**27**)

In 2016, Tang et al. [56] isolated three new carboxyxanthones, oliganthic acid A (**25**), oliganthic acid B (**26**), and (±)-oliganthic acid C (**27**) (Figure 3), from the leaves of *Garcinia oligantha*. The cytotoxicity activity was evaluated against A549, HepG2, HT-29, PC3, and HL-7702 human cancer cell lines; however, no activity against these cell lines was observed.

### 2.3. Caged Carboxyxanthone Derivatives

#### 2.3.1. Gambogic Acid (**28**) and Analogues (**29**–**70**)

**Figure 4:**
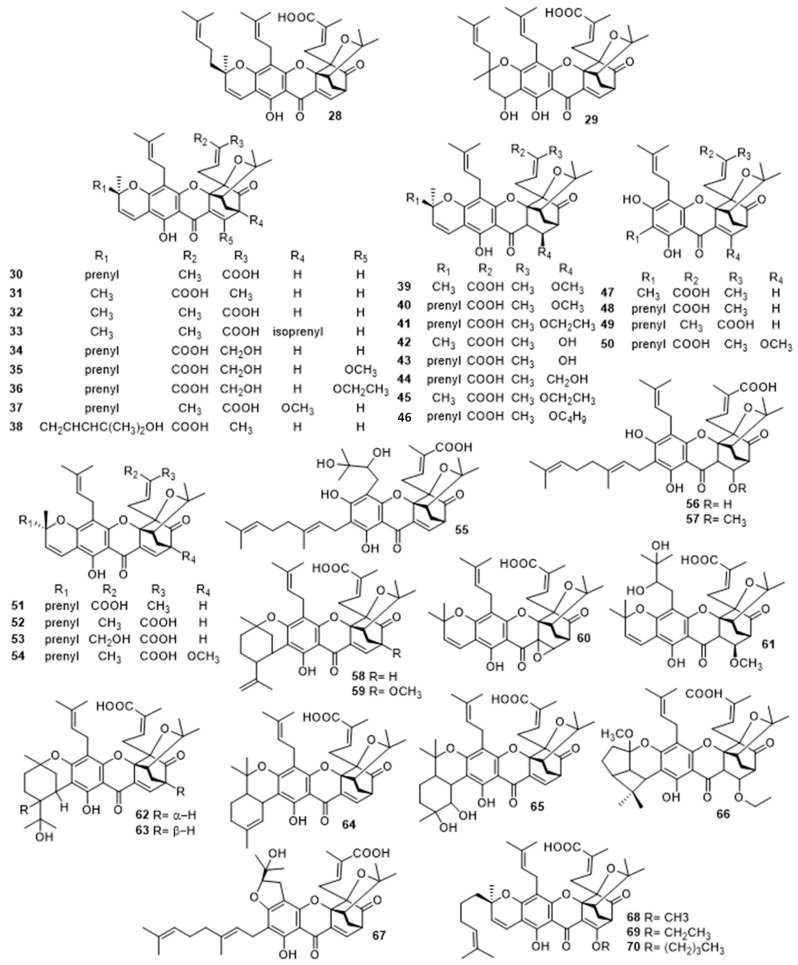
Structures of gambogic acid (**28**) and analogues (**29**–**70**).

Gambogic acid (**28**) and neogambogic acid (**29**) were firstly isolated, in 1984, by Lu et al. [57] from *Garcinia hanburyi*. Since then, several studies regarding the isolation and biological activity evaluation of gambogic acid (**28**) and its analogues (**29**–**70**) (Figure 4) have been published [58,59,60,61,62,63,64,65,66,67,68,69,70,71,72,73]. In 1993, Lin et al. [58] reported the isolation of isogambogic acid (**30**). In 1996, Asano et al. [59] reported the isolation of five additional caged carboxyxanthone derivatives from the gamboge resin of *Garcinia hanburyi*, including the previously reported gambogic acid (**28**), as well as the morellic (**31**), moreollic (**39**), gambogenic (**47**) and gambogellic (**58**) acids [59].

For the past 17 years, several research groups have reported the isolation of novel caged prenylated carboxyxanthones, analogues of gambogic acid, from the leaves, resin and fruits of *Garcinia hanburyi* and *Garcinia morella*, including, isomorellic acid (**32**) [60], 7-isoprenylmorellic acid (**33**) [60], 30-hydroxygambogic acid (**34**) [65], 10-methoxygambogic acid (**35**) [67], 10-ethoxygambogic acid (**36**) [67], 7-methoxygambogic acid (**37**) [68], oxygambogic acid (**38**) [68], gambogic acids A and B (**40** and **41**) [63], 8,8a-dihydro-8hydroxymorellic acid (**42**) [68], 8,8a-dihydro-8-hydroxygambogic acid (**43**) [68], garcinolic acid (**44**) [69], 10α-ethoxy-9,10-dihydromorellic acid (**45**) [69], 10α-butoxygambogic acid (**46**) [72], gaudichaudic acid (**48**) [63], isogambogenic acid (**49**) [63], 10-methoxygambogenic acid (**50**) [67], epigambogic acid (**51**) [64], 30-hydroxyepigambogic acid (**52**) [65], epiisogambogic acid (**53**) [66], 7-methoxyepigambogic acid (**54**) [68], 12-hydroxygambogefic acid (**55**) [70], 8,8a-dihydro-8-hydroxygambogenic acid (**56**) [68], 10-methoxygambogenic acid (**57**) [69], 7-methoxygambogellic acid (**59**) [68], 8,8a-epoxymorellic acid (**60**) [62], hanburinone (**61**) [61], gambogollic acid (**62**) [71], epigambogollic acid (**63**) [71], gambogefic acid (**64**) [68], 22,23-dihydroxydihydrogambogenic acid (**65**) [70], gambogic acid C (**66**) [72], gambogenific acid (**67**) [68] and epigambogic acids A, B and C (**68**–**70**) [72]. All these compounds were subjected to bioactivity assays and, it is important to highlight their overall cytotoxic activities against several cell lines including P-388, KB, Col-2, BCA-1, LU-1, ASK, K-562/ADR and K-562/S [62,63,74]. Anti-HIV-1 activity of gambogic acid (**28**) and morellic acid (**31**), by inhibiting the HIV-1 reverse transcriptase enzyme [62] and the antiatherosclerosis activity of gambogic acid (**28**) via inhibition of vascular smooth muscle cell proliferation were also significant [75]. The isolation and biological activity evaluation of these compounds have been extensively reviewed by several groups [76,77,78,79,80].

#### 2.3.2. Gaudichaudiic Acids A–I (**71**–**79**)

**Figure 5:**
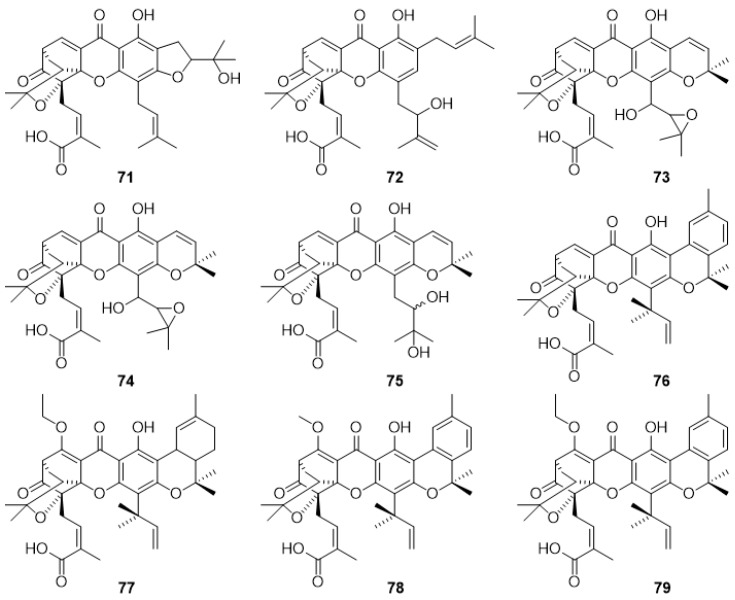
Structures of gaudichaudiic acid A–I (**71**–**79**).

In 1998, Cao et al. [81] isolated a set of five caged carboxyxanthone derivatives from the leaf extract of *Garcinia gaudichaudii*, namely, gaudichaudiic acids A–E (**71**–**75**). Later, in 2000, other caged carboxyxanthone derivatives, gaudichaudiic acids F–I (**76**–**79**), were reported by Xu et al. [82]. In both studies, compounds **71**–**79** (Figure 5) were tested for cytotoxicity against several cell lines, including P388/DOX and Messa [82], P388 [81,82], WEHI1640, MOLT4, HePG2, and LL/2 [81]. It was found that all compounds showed cytotoxic activity against P388 cell line. Gaudichaudiic acids A–E (**71**–**75**) were also active against WEHI1640, MOLT4 and LL/2, while only gaudichaudiic acids A (**71**) and E (**75**) showed activity against the HePG2 cell line [81]. Regarding gaudichaudiic acids G–I (**77**–**79**), they were cytotoxic against P388/DOX and Messa cell lines [82].

#### 2.3.3. Scortechinones (**80**–**90**)

**Figure 6:**
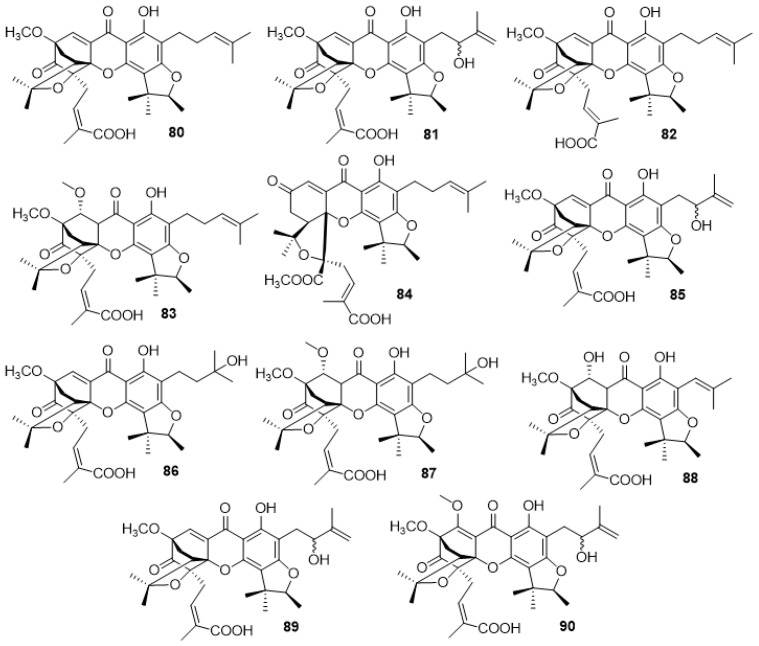
Structure of scortechinones **80**–**90**.

The isolation of caged carboxyxanthones was primarily achieved by Rukachaisirikul and colleagues [83,84,85,86], from several plant parts of *Garcinia scortechinii*. In 2000, the same group reported the isolation of scortechinones B (**80**), and C (**81**) (Figure 6), from the twigs of *Garcinia scortechinii* [83]. These compounds were tested for antimicrobial activity against methicillin-resistant *Staphylococcus aureus* (MRSA SK1), and both showed good antibacterial activity [83]. Later, in 2003, three new carboxylated scortechinones were isolated from the latex of *Garcinia scortechinii*, namely, scortechinones F (**82**), G (**83**) and K (**84**), along with the previously mentioned scortechinone B (**80**) [84]. A year later, four new carboxylated scortechinones (M–P) (**85**–**88**) (Figure 5) were isolated from the bark stem of *Garcinia scortechinii*, along with scortechinones **80**–**84** [85]. Scortechinones C (**81**) and M (**85**) were identified as having identical structures; however, due to the difference in their optical rotation values, scortechinone M (**85**) was identified as a C-11 epimer of scortechinone C (**81**) [85]. All the isolated scortechinones were tested for antibacterial activity against two strains of *Staphylococcus aureus*, namely ATCC25923 and MRSA SK1 [83]. In this study, the antibacterial activities of scortechinones B (**80**), and C (**81**) against MRSA SK1 [83], as well as against the ATCC25923 strain was confirmed. Regarding scortechinones F (**82**), G (**83**), and K (**84**), it was found that these compounds were active against both *Staphylococcus aureus* strains [84]. The best minimum inhibitory concentration (MIC) indices were achieved by Scortechinone F (**82**). Scortechinones M–P (**85**–**88**) presented good antibacterial activity results overall, with scortechinone P (**88**) showing the best MIC indices for both strains [85].

In 2005, two more caged carboxylated scortechinones were isolated from the fruits of *Garcinia scortechinii*, specifically scortechinones *R* (**89**) and *S* (**90**), (Figure 6) [86]. These new scortechinones (**89**–**90**) were tested against MRSA SK1, showing good antibacterial activity [86].

### 2.4. Carboxyxanthone Derivatives Bound or Fused to Polysubstituted Oxygenated Heterocycles

#### 2.4.1. Vinaxanthone 411F (**91**) and Analogues (**92**–**95**)

**Figure 7:**
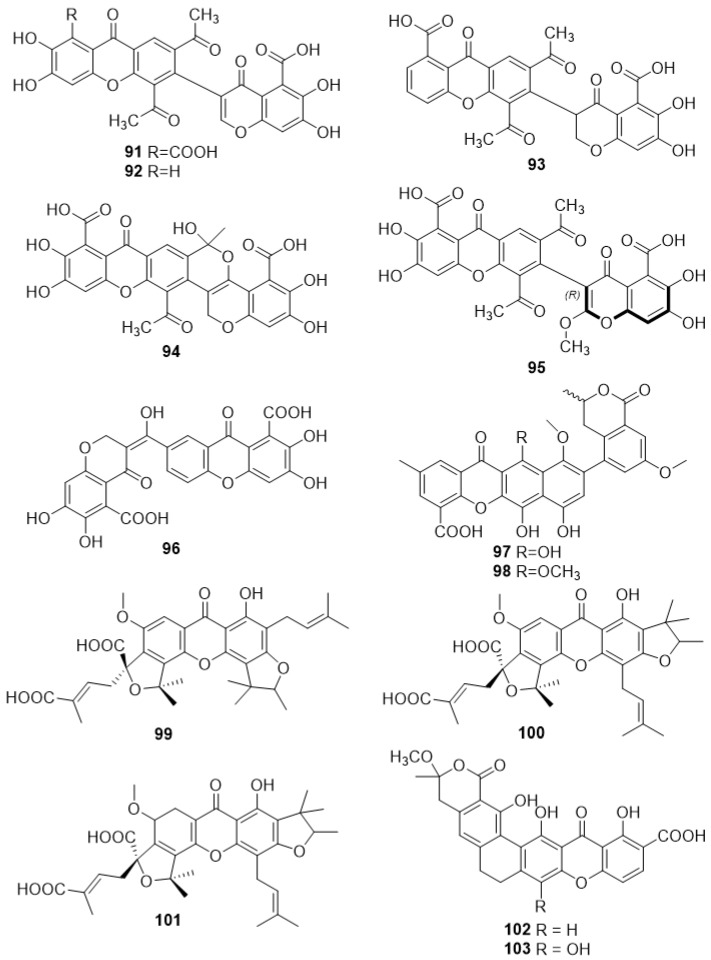
Structures of carboxyxanthone derivatives bound or fused to polysubstituted oxygenated heterocycles (**91**–**103**).

Vinaxanthone 411F (**91**) (Figure 7) was firstly isolated from *Penicillium vinaceum NR6815*, by Aoki et al. [87], in 1991, being identified as a novel phospholipase C selective inhibitor of murine colon 26 adenocarcinoma and murine fibroblasts NIH3T3. Three years later, it was found that vinaxanthone 411F (**91**) also interact with multiple sites of CD4 cells, inhibiting anti-Leu3a and HIV gp120 binding to human CD4 cells, as well as antigen-induced T-cell proliferation of CD4+ [88]. In the same year, three new vinaxanthone analogues were isolated from *Penicillium glabrum*, specifically vinaxanthones 411P (**92**), 411J (**93**), and 2383 (**94**), the cyclized form of 411J (Figure 7) [89]. In 2008, another vinaxanthone analogue, comprising axial chirality, (a*R*)-2′-methoxyvinaxanthone (**95**), (Figure 7), along with the previously reported vinaxanthones **91** and **92**, were isolated from a strain of *Penicillium vinaceum* [90]. In this study, vinaxanthone 411F (**91**), vinaxanthone 411J (**93**) and (a*R*)-2′-methoxyvinaxanthone (**95**) exhibited significant growth inhibition of crown gall tumors on *Agrobacterium tumefaciens* cultures [90]. Recently, other activities were reported for vinaxanthone **91**, such as inhibition of the bacterial enzyme enoyl-ACP reductase (FabI) from *S. aureus*, as well as a growth inhibition of two resistant strains, namely *methicillin-resistant* and *quinolone-resistant S. aureus* [91].

#### 2.4.2. Xanthofulvin (**96**)

In 1993, the pharmaceutical company *Hoffmann-La Roche AG*, in the person of Dr. Masubuchi, filed a patent on the isolation of a new carboxyxanthone, xanthofulvin (**96**) (Figure 7), from cultures of *Eupenicillium sp. NR7125* [92]. This compound (**96**) was found to have good inhibitory activity against the enzyme chitin synthase [92]. A decade later, in 2003, Kumagai et al. [93] isolated compound **96** from cultures of *Penicillium sp. SPF-3059*, and demonstrated that it also exhibited semaphorin inhibitory activity. In the same year, Kikuchi et al. [94] and Kaneko et al. [95] reported that xanthofulvin (**96**) was the first described Sema3A inhibitor in both in vitro and in vivo studies promoting spinal cord regeneration. Recently, it was evaluated for inhibition of cysteine synthase enzyme by Mori et al. [96] showing inhibitory activity against both EhCS1 and EhCS3. Recently, the mechanism of action of xanthofulvin (**96**) and vinaxanthone (**91**) for inhibition of Sema3A have been described [97].

#### 2.4.3. 6,7,11-Trihydroxy-10-Methoxy-9-(7-Methoxy-3-Methyl-1-Oxoisochroman-5-yl)-2-Methyl-12-Oxo-12*H*-Benzo[b]Xanthene-4-Carboxylic Acid (**97**) and 6,7-Dihydroxy-10,11-Dimethoxy-9-(7-Methoxy-3-Methyl-1-Oxoisochroman-5-yl)-2-Methyl-12-Oxo-12*H*-Benzo[b]Xanthene-4-Carboxylic Acid (**98**)

In 2012, Omolo et al. [98] isolated two new carboxyxanthones, 6,7,11-trihydroxy-10-methoxy-9-(7-methoxy-3-methyl-1-oxoisochroman-5-yl)-2-methyl-12-oxo-12*H*-benzo[b]xanthene-4-carboxylic acid (**97**) and 6,7-dihydroxy-10,11-dimethoxy-9-(7-methoxy-3-methyl-1-oxoisochroman-5-yl)-2-methyl-12-oxo-12*H*-benzo[b]xanthene-4-carboxylic acid (**98**) (Figure 7), from the tubers of *Pyrenacantha kaurabassana*. Their activity against an HIV strain via the deCIPhR assay was evaluated demonstrating that both compounds showed moderate anti-HIV activity; however, low selectivity indices were observed, concluding that they were not effective as anti-HIV entry inhibitors [98].

#### 2.4.4. Scortechinones V (**99**), W (**100**) and X (**101**)

Scortechinones V (**99**), W (**100**), and X (**101**) (Figure 7) were isolated from the fruits of *Garcinia scortechinii*, together with the previously described caged scortechinones *R* (**89**) and *S* (**90**) (Figure 6) [86]. These carboxylated derivatives presented antibacterial activity against MRSA SK1, especially scortechinone W (**100**), showing the lowest MIC value (52.8 µM) [86].

#### 2.4.5. Dehydrocitreaglycon A (**102**) and Citreaglycon A (**103**)

In 2012, Liu et al. [99] isolated two new carboxyxanthones, dehydrocitreaglycon A (**102**) and citreaglycon A (**103**) (Figure 7), from marine-derived *Streptomyces caelestis*. These two compounds showed antibacterial activity against *S. haemolyticus*, *S. aureus* and *Bacillus subtillis* [99,100].

## 3. Synthetic Carboxyxanthone Derivatives

Michael and Kostanecki introduced one of the first methods for the synthesis of xanthones, which involved the distillation of a mixture of a phenol, *O*-hydroxybenzoic acid, and acetic anhydride [101,102]. Since then, several other routes affording higher yields and less drastic experimental conditions have been developed [103,104,105,106,107,108,109,110].

**Figure 8:**
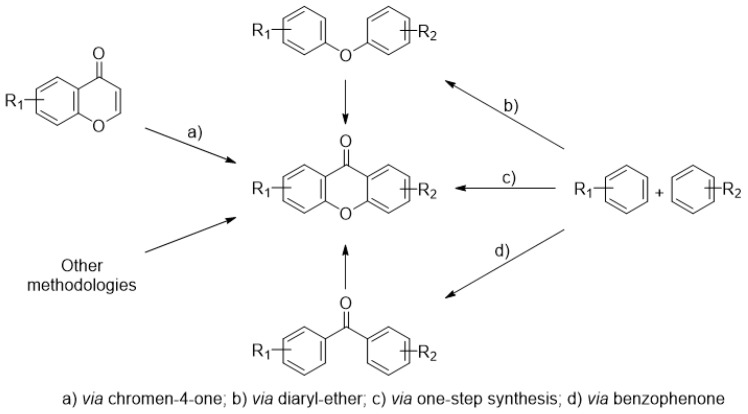
Commonly used synthetic routes of xanthones.

In general, four methods can be applied for the synthesis of simple xanthones: Grover, Shah and Shan method, in one step reaction, synthesis via benzophenone and diaryl ether intermediates, which overcome the limitations of one-step methods [17,18], and synthesis via chromen-4-one derivatives [111] (Figure 8). For the synthesis of carboxylated xanthone derivatives any of these methods can be applied if using suitable building blocks.

### 3.1. DMXAA *(**2**)*, XAA *(**104**)* and Analogues *(**105**–**161**)*

Among the synthetic carboxyxanthone derivatives, DMXAA (5,6-dimethylxanthone-4-acetic acid, Vadimezan, ASA404, **2**, Figure 1) aroused much interest in the scientific community due to its remarkable pharmacological profile. Several reviews can be found in the literature focused on DMXAA (**2**), mainly highlighting its antitumor activity [36,112,113,114,115,116,117,118,119,120,121]. DMXAA (**2**) selectively attacks established tumor blood vessels through induction of apoptosis in tumor vascular endothelial cells [122,123], causing vascular collapse and hemorrhagic necrosis, and expanding tumor hypoxia [124,125]. It has inductive effects on different cytokines, chemokines, and vasoactive factors [126,127,128], which interact with tumor endothelial cells resulting in hemorrhagic tumor necrosis. It also induces nitric oxide [129,130,131], serotonin [132,133], and nuclear factor κB [134,135]. In addition to antitumor activity, other activities have been reported for DMXAA **(2**), including antiviral [136], antiplatelet and antithrombotic [137]. In phase I/II clinical trials, DMXAA (**2**), in combination with standard anticancer agents, showed promising results for the treatment of non–small-cell lung cancer [138,139,140,141,142]; however, in two large-scale phase III clinical trials the combination of DMXAA (**2**) with other anticancer drugs failed to increase their efficacy [143].

**Figure 9:**
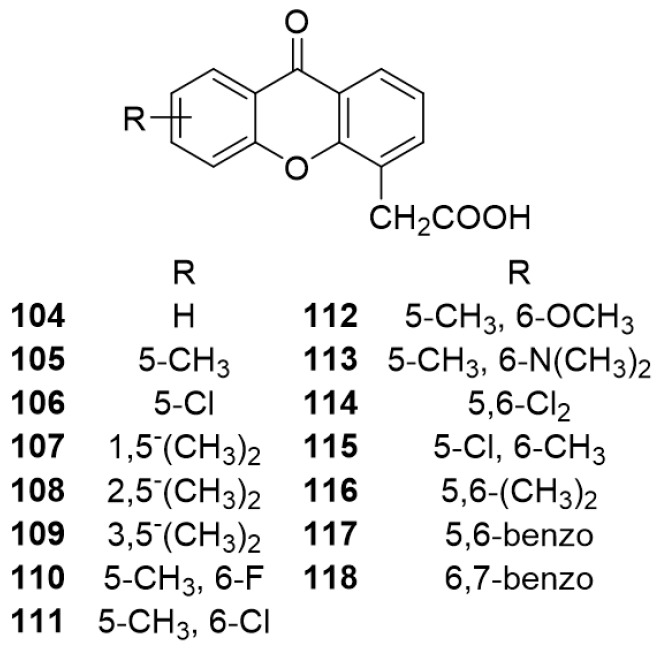
Structure of XAA (**104**) and analogues **105**–**118**.

This carboxyxanthone derivative (**2**) was discovered, in 1991, in a structure-activity relationship study using diverse xanthenone-4-acetic acid (XAA, **104**) analogues (**105**–**118**) of a flavone acetic acid drug (Figure 9) [144]. Analogues **107**–**109** comprising only one substituent in each aromatic ring of xanthone scaffold, were synthesized by coupling sodium salts of 2-iodo-3-methylbenzoic acid with a suitable methyl-substituted 2-hydroxyphenylacetic acid, using *tris*-[2-(2-methoxyethoxy)ethyl]amine as catalyst. Then, an acid-catalyzed cyclodehydration of the obtained diacids was carried out [144]. The same route was used for analogues **110**–**111** and **114**–**118**, including DMXAA (**2**), by coupling salts of 2-hydroxyphenylacetic acid with appropriate disubstituted 2-iodobenzoic acids. For the analogues **112**–**113**, a nucleophilic displacement of chlorine from 6-chloro-5-methyl-9-oxo-*9H*-xanthene-4-acetic acid with methoxide and dimethylamine, respectively, was performed [144].

In 2002, an improved synthesis of DMXAA (**2**) was developed by optimization of the synthesis of the key intermediate 3,4-dimethylanthranilic acid via nitration of 3,4-dimethylbenzoic acid and separation by crystallization [145]. A higher overall yield was obtained from 3,4-dimethylbenzoic acid, specifically 22%. Seven years later, a new short and efficient synthesis of DMXAA (**2**) was reported using 3,4-dimethylbenzoic acid as starting material [146]. The synthetic pathway comprises of four steps, being the key steps the dibromination of 3,4-dimethylbenzoic acid, followed by the regioselective coupling with 2-hydroxyphenylacetic acid and further cyclodehydration, in an overall yield of 51%.

**Figure 10:**
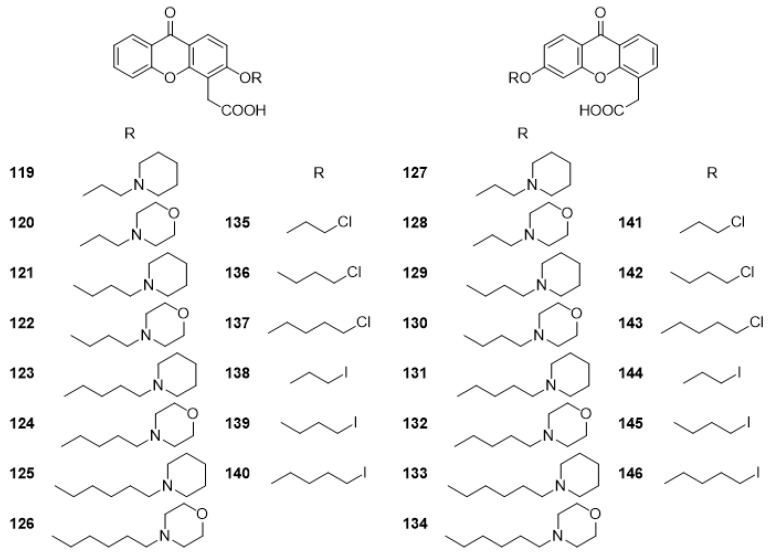
Structure of DMXAA analogues **119**–**146**.

From a biological activity perspective, it is evident that DMXAA (**2**) may be a useful scaffold for the development of other bioactive compounds and, over the years, several analogues and derivatives have been developed. In 2006, Gobbi et al. [147], synthetized several carboxylated DMXAA (**2**) analogues (**119**–**134**) with potential antitumoral activity (Figure 10). The synthesis was performed through a multi-step pathway by derivatization of 4-allyl-3-hydroxy-9*H*-xanthen-9-one. All compounds were tested for antiproliferative activity towards human ovarian adenocarcinoma 2008 cell line, and cisplatin-resistant subline C13* [147]. It was found that compounds **119** and **128** presented good ability to inhibit 2008 cell line [148]. Most of the other compounds only presented cytotoxic activity at the highest tested concentration [147].

In the same study, Gobbi et al. [147] also described another 12 XAA derivatives (**135**–**146**) (Figure 10), specifically the intermediates for synthesis of the analogues **119**–**134**; however, they were not tested for cytotoxic activity.

**Figure 11:**
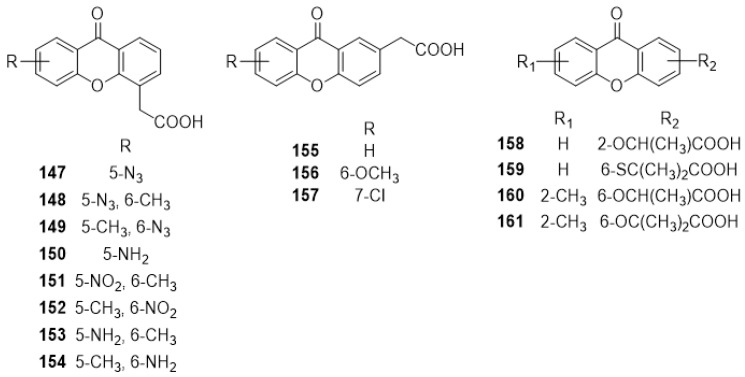
Structures of DMXAA analogues **147**–**161**.

In 2007, eight new analogues of DMXAA (**2**) and XAA (**104**) bearing azido, nitro and amino moieties, compounds **147**–**154** (Figure 11), were reported by Palmer [148]. All compounds were tested for their cytotoxicity on HECPP murine endothelial cells, as well as their ability to induce hemorrhagic necrosis in mice with colon 38 tumors [148]. It was found that compounds **147** and **148** caused profound necrosis on the tested tumors, when compared to the carboxyxanthone derivative **2** [148]. Compound **147** was able to bind specifically to cellular proteins through photoreaction, which could be a useful tool to identify the receptors of DMXAA (**2**) [148]. In 2009, Marona et al. reported the synthesis of seven new analogues (**155**–**161**) (Figure 11) of DMXAA (**2**), with weak cytotoxicity against J7774A.1 cells [149].

Moreover, additional efforts aiming to identify derivatives with improved activity than DMXAA (**2**) are under investigation. Recently, DMXAA-pyranoxanthone hybrids were reported to enhance inhibition activity against human cancer cells with multi-target functions [150].

### 3.2. 9-Oxo-9H-Xanthene-2-Carboxylic Acid *(**162**)* and Analogues *(**163**–**284**)*

#### 3.2.1. Synthesis

The synthesis of 9-oxo-9*H*-xanthene-2-carboxylic acid (**162**) was first reported by Anschutz et al. [151], in 1925, from 2-methylphenylsalicilate. Later, in 1960, El Abbady et al. [152], described its synthesis through oxidation of γ-oxo-γ-2-xanthenylbutyric acid. In 1977, Graham and Lewis [153], described other synthetic strategy, via benzophenone intermediate, through reaction of 2-methoxybenzoic acid with methyl 4-hydroxybenzoate. Later, in 1998, the same carboxyxanthone (**162**) was synthesized by Pickert and Frahm [154], via diaryl ether intermediate, using Ullman coupling reaction of 2-chlorobenzoic acid with 4-hydroxybenzoic acid.

Several analogues of 9-oxo-9*H*-xanthene-2-carboxylic acid (**162**) have been synthesized through the years, holding different patterns of substitution (Table 1) [151,153,154,155,156,157,158,159,160,161,162,163,164,165,166]. The synthetic methodologies used to obtain these analogues were via diaryl ether and benzophenone intermediates, and through the derivatization of xanthones as building blocks. In 1972, Pfister et al. [155], synthesized various analogues (**163**–**184**) with potential antiallergic activity. 1-Methoxy-9-oxo-9*H*-xanthene-2-carboxylic acid (**163**) was obtained through Friedel-Crafts acylation of 1-hydroxyxanthone and further methylation followed by an oxidation with NaBrO [155]. Xanthone-2-carboxylic acids **164**–**178** were synthesized via diaryl ether intermediates, by Ullmann coupling reactions between an aryl halide and a phenol followed by intramolecular electrophilic cyclization, using polyphosphoric acid as catalyst [155]. The total synthesis of carboxyxanthone derivatives **166** and **169** were also reported by our group, being the methodologies improved in order to decrease reaction time and to increase the final yield [167].

7-Chloro-9-oxo-9*H*-xanthene-2-carboxylic acid (**178**) was also synthesized by Graham and Lewis, in 1977, via benzophenone intermediate, through the reaction of 5-chloro-2-mehoxybenzoic acid with methyl 4-hydroxybenzoate [153]. 7-Hydroxy-9-oxo-9*H*-xanthene-2-carboxylic acid (**179**) was obtained through ether cleavage of 7-methoxy-9-oxo-9*H*-xanthene-2-carboxylic acid (**168**) using HBr in acetic acid, and analogues **180**–**184** through alkylation of **168** with the corresponding haloalkane [155]. The synthesis of analogues **186**–**205** was reported by Bristol et al., in 1978, through alkylation of methyl 7-hydroxy-9-oxo-9*H*-xanthene-2-carboxylate with epichlorohydrin, followed by reaction of the obtained epoxide with a suitable mercaptide or alkoxide, in basic conditions, and further hydrolysis of the ester [157].

In 1978, a series of other 9-oxo-9*H*-xanthene-2-carboxylic acid analogues (**206**–**231**) were specifically developed for antiallergic activity, by Pfister et al. [158], using different methodologies. Analogues **206**–**210** were obtained using carboxyxanthone **162** as a building block to obtain xanthene-2-carboxylic acid through a Huang-Minlon reduction, followed by esterification of the carboxylic acid, and Friedel-Crafts acylation with an acyl halide. The obtained compound was then oxidized with Jones reagent, and the saponification of the ester provided the desired compounds [158]. 7-Mercapto-9-oxo-9*H*-xanthene-2-carboxylic acid (**211**) was prepared through derivatization of methyl 7-hydroxy-9-oxo-9*H*-xanthene-2-carboxylate with dimethylcarbamothioic chloride, followed by thermal rearrangement and base hydrolysis. Compound **211** was used as precursor for synthesis of analogues **212**–**216**, through alkylation with MeI or *i*-C_3_H_7_Br, and further oxidation and base hydrolysis to afford compounds **212** and **213**, or simply base hydrolysis to obtain compounds **214** and **215** [158]. Oxidation of 7-(methylthio)-9-oxo-9*H*-xanthene-2-carboxylic acid (**214**) with hydrogen peroxide in acetic acid gave 7-(methylsulfonyl)-9-oxo-9*H*-xanthene-2-carboxylic acid (**216**) [158]. Ullman coupling reactions between dimethyl 4-bromoisophthalate and several phenols were performed for the synthesis of six diaryl ether intermediates that, after saponification and intramolecular electrophilic cyclization, afforded compounds **217**–**223** [158]. 5-Methoxy-7-(methylthio)-9-oxo-9*H*-xanthene-2-carboxylic acid (**223**) was used as precursor for synthesis of analogues **224**–**231** through *O*-demethylation of the methoxy group at 5-position of xanthone scaffold, followed by esterification of the carboxylic acid using suitable haloalkane, and further saponification [158].

In 1979, Barnes et al. [159], described the synthesis of several analogues bearing a sulphur-based moiety at 7-position of xanthone scaffold (methylthio, methylsulfinyl, and *S*-methylsulfonimidoyl groups). Analogues **232**–**236** and **233**–**235** were synthesized via diaryl-ether intermediate. Through Ullmann coupling reaction between a methyl 4-bromoisoftalate and 4-mercaptophenol, 2-hexyl-4-mercaptophenol, or 4-mercapto-2-(pentyloxy)phenol, followed by ester hydrolysis, and further intramolecular cyclization using polyphosphoric acid as catalyst, compounds **232**–**234** were obtained [159]. The carboxylic acid group of these compounds was then protected through esterification, and oxidation of the methylthio group was performed to afford the analogues **235**, **236** and **228**, after saponification, [158,159]. The methyl esters of these compounds were further reacted with sodium azide and polyphosphoric acid to give compounds **237**–**239**, post saponification. Several *N*-substituted sulfoximidoxanthonecarboxylic acids (**240**–**246**) were also obtained through the reaction of methyl esters of **237** and **238** with a suitable reagent, followed by ester hydrolysis [159]. Analogue **247** was prepared by the same methodology; however, the compounds used for the reaction was 7-(methylthio)-9-oxo-9*H*-xanthene-2-carboxylic acid (**232**) [159].

Pfister and Wymann [161], in 1980, reported several 7-sulfamoyl-9-oxo-9*H*-xanthene-2-carboxylic acid analogues (**248**–**267**) as potential aldose reductase inhibitors. The synthesis of these compounds was achieved through three different pathways [161]. First, a chlorosulfonation of 9-oxo-9*H*-xanthene-2-carboxylic acid (**162**) with chlorosulfonic acid was performed to afford 7-(chlorosulfonyl)-9-oxo-9*H*-xanthene-2-carboxylic acid (**248**) and then reacted with NaOH or an amide to give analogues **249**–**261** [161]. The second pathway consisted in a reaction of 2-bromoethanol with the thiol group of 7-mercapto-9-oxo-9*H*-xanthene-2-carboxylic acid (**211**) to afford 7-((hydroxyethyl)thio)-9-oxo-9*H*-xanthene-2-carboxylic acid (**262**), followed by protection of the acid group through esterification with methyl iodide. The methyl ester of **262** was then oxidized to obtain analogues **263** and **264**, after ester hydrolysis [161]. 7-((2-Methoxyethyl)sulfinyl)-9-oxo-9*H*-xanthene-2-carboxylic acid (**265**) was achieved by reaction of methyl iodide with the 2-hydroxyethylthio moiety of the methyl ester of **262**, followed by ester hydrolysis [161]. Finally, analogue **266** was obtained through a catalytic hydrogenation of sodium 7-acetyl-9-oxo-9*H*-xanthene-2-carboxylate, and **267** by formation of a methyl ether with methyl iodide in acidic conditions [161]. Two years later, the same group developed two more analogues (**268** and **296**), by Ullmann coupling reaction of methyl 4-bromoisoftalate with 2,4-diisopropylphenol and 2,4-di-*tert*-butylphenol, respectively, followed by intramolecular electrophilic acylation using polyphosphoric acid [162].

In 1993, Sawyer and coworkers [163,164] were able to synthesize the analogues **270**–**273**, as potential antagonists for leukotriene B_4_ receptor, through Ullmann coupling reaction of suitable phenols and aryl bromides, followed by cyclization [163]. Analogue **274** was obtained through reaction of methyl 5-(3-ethoxy-3-oxopropyl)-6-hydroxy-9-oxo-9*H*-xanthene-2-carboxylate with 4-(3-chloropropoxy)-5-ethyl-4′-fluoro-2-phenoxy-1,1′-biphenyl, followed by saponification [164].

Pickert and Frahm described, in 1998, a series of carboxy- and dicarboxyxanthone derivatives bearing nitro and amino groups (**275**–**280**) [154]. These compounds were synthesized via diaryl ether intermediate by reaction of a series of benzoyl halides and phenols. In 2001, Fonteneau et al. [166] reported the synthesis of analogues **281**–**283**, through reaction of 2,6-dihydroxybenzoic acid with 5-methyl resorcinol to give 1-hydroxy-3-methyl-9-oxo-9*H*-xanthene, followed by suitable derivatization (analogues **281**–**282**), and through reaction of 2,6-dihydroxybenzoic acid with phloroglucinol, followed by esterification and deprotection (analogue **283**) [166]. In 2003, Hernández et al. [168] synthesized a novel carboxyxanthone (**284**), via diaryl ether intermediate by reaction of 4-bromo-5-nitroisophthalic acid with potassium 4-(*tert*-butyl)-2-nitrophenolate.

It is important to emphasize that, in our group, carboxyxanthone derivative **169** has been used as a suitable building block for the synthesis of several chiral derivatives [167,169] with high enantiomeric purity [170,171,172]. Some chiral derivatives showed interesting growth inhibitory activity on A375-C5, MCF-7 and NCI-H460 human tumor cell lines [167], ability to block sciatic nerve transmission [169] and inhibit cyclooxygenases 1 and 2 enzymes [173]. Some of them were also promising chiral selectors in liquid chromatography enantioseparation [21,22].

#### 3.2.2. Biological Activities

In general, 9-oxo-9*H*-xanthene-2-carboxylic acid (**162**) and analogues **163**–**284** have been studied for antiallergic activity [155,156,157,158,159]. Some of them have also been tested for inhibitory activity against aldose reductase and as antagonists of leukotriene B_4_ receptor [161,163].

Carboxyxanthone derivative **162** presents relatively low antiallergic activity, in rat passive cutaneous anaphylaxis (PCA) assay, when compared with disodium cromoglycate [155,158]. In general, for analogues of **162** it was found that, the presence of small groups in 5- and 7-positions of xanthone scaffold, often increase the activity, while the presence of bulky groups have the opposite effect [155,158,160]. In fact, several 5-substituted (**167**, **176**, **184**, **212**, **214**, **216**, **224**–**231**, **233**–**234**, **236** and **238**–**239**) and 7-substituted (**168**, **171**, **173**–**174**, **182**, **185**, **192**, **206**, **232**, **235** and **237**) compounds exhibited higher antiallergic activity, when compared to **162**, being some compounds (**173**–**174**, **182**, **192**, **237** and **238**) orally active [155,156,157,158,159,160].

Inhibitory activity against aldose reductase enzyme was evaluated for compound **162** and analogues **249**–**267** [161]. 7-(*N*,*N*-Dimethylsulfamoyl)-9-oxo-9*H*-xanthene-2-carboxylic acid (**252**) was proved to be a good noncompetitive inhibitor of the enzyme; while 7-(*N*-(2-hydroxyethyl)-*N*-methylsulfamoyl)-9-oxo-9*H*-xanthene-2-carboxylic acid (**259**) presented the higher potency of all tested compounds [161].

Compounds **270**–**274** were studied as antagonists of leukotriene B_4_ receptor (LTB_4_) [163,164]. These compounds were shown to be, in general, good antagonists of LTB_4_ by blocking the up-regulation of the CD11b/CD18 receptor, being compounds **271**, **272** and **274** the most active LTB_4_ antagonists. It is also important to highlight that compound **274** presented strong binding abilities to human neutrophils and guinea pig lung membranes, being one of the most potent antagonists [163,164].

### 3.3. Other 9-Oxo-9H-Xanthene Carboxylic Acid Derivatives *(**285**–**338**)*

#### 3.3.1. Synthesis

The synthesis of 9-oxo-9*H*-xanthene-1-carboxylic acid (**285**), 9-oxo-9*H*-xanthene-3-carboxylic acid (**286**) and 9-oxo-9*H*-xanthene-4-carboxylic acid (**287**) (Table 2), was described for the first time by Anschutz et al. [151], in 1925, and were obtained through the intramolecular acylation of 2-(3-carboxyphenoxy)benzoic acid or 2,2′-oxydibenzoic acid. In 1998, Pickert and Frahm [154], described their synthesis via diaryl ether intermediate, by Ullmann coupling reaction of an aryl halide and a phenol.

El Abbady [152] reported, in 1960, the synthesis of carboxyxanthone derivative **288** (Table 2) through oxidation of 4-oxo-4-(9*H*-xanthen-2-yl)butanoic acid with potassium permanganate in acetone. In 1990, Sato et al. [174] reported the synthesis of several new carboxyxanthone derivatives (**289**–**320**). Compounds **289**–**306** (Table 2) were synthesized via benzophenone intermediate through reaction of 2-fluorobenzoyl chlorides or 2-chlorobenzoyl chlorides with 5-substituted-1,3-dimethoxybenzene, 2-substituted-1,3-dimethoxybenzene or 1-substituted-2,4-dimethoxybenzene, followed by basic etherification reaction to give 3-methoxy-9*H*-xanthen-9-one derivatives. Then, a reaction with ethyl 2-bromoacetate and further saponification were carried out [174]. Carboxyxanthone derivatives **307**–**320** were obtained through reaction of 3-hydroxy-9*H*-xanthen-9-one derivatives with 3-bromoprop-1-ene followed by reaction with *N*-methylaniline or *N*-ethylaniline to give both 4-allyl-3-hydroxy-9*H*-xanthen-9-one and 2-allyl-3-hydroxy-9*H*-xanthen-9-one derivatives, that through oxidation with *m*-chloroperbenzoic acid followed by Jones oxidation, afforded compounds **307**–**315** and **316**–**320**, respectively (Table 2) [174].

Jackson et al. [163] described in 1993, the synthesis of carboxyxanthone derivatives **321** and **322** (Table 2) via diaryl ether intermediate through Ullmann coupling reaction of suitable phenols and aryl bromides, followed by cyclization [163]. The synthesis of compounds **324**–**332** (Table 2) were reported in 1998, by Pickert et al. [154], through the same synthetic pathway as described for compounds **276**–**281**. Recently, Zelaszczyk et al. [175] synthesized carboxyxanthone derivatives **333**–**338** (Table 2) though derivatization of the previously described 3-hydroxyxanthones with sodium chloroacetate or ethyl 2-bromopropanoate followed by ester hydrolysis.

In our group, carboxyxanthone derivative **289** has been used as a building block to obtain diverse chiral derivatives with potential biological activities [167,169,173], as well as chiral selectors for analytical liquid chromatography application [21,22].

#### 3.3.2. Biological Activities

Carboxyxanthone derivatives **289**–**320** were screened for their potential diuretic and uricosuric activities in rats and compared with tienilic acid and indacrinone [174]. These compounds presented, in general, similar or more potent, diuretic activities when compared to tienilic acid [174]. Some compounds (**299**, **301**, **304**, **306**, **310**, **312**, and **320**) also showed balanced diuretic and uricosuric activities, with compound **301** presenting better balanced activities when compared with indacrinone [174]. Carboxyxanthone derivatives **321** and **320** were evaluated as antagonists of leukotriene B4 receptor [163]. Compounds **333**–**338** were tested for analgesic, anti-edema and ulcerogenic activities [175]. Both compounds **337** and **338** exhibited promising anti-inflammatory activity with compound **338** also showing excellent analgesic activity. 

## 4. Conclusions

During several years, diverse carboxyxanthone derivatives have been obtained either from natural sources or by synthetic methods. Nature afforded more complex structures, but synthetic methodologies could furnish a large variety of carboxyxanthone derivatives for biological activity and structure-activity relationship studies, enlarging the chemical/biological space. For the synthesis of carboxylated xanthone derivatives, diverse methods can be applied if using suitable building blocks. The biological and pharmaceutical significance of these compounds in different areas have been highlighted in this review. Some of them revealed promising activities including antibacterial, antifungal, antiviral, antitumor, antiallergic, anti-inflammatory, diuretic and uricosuric activities as well as inhibitory activity against aldose reductase and as antagonists of leukotriene B4 receptor. Their application as suitable chemical substrates to obtain new bioactive derivatives was also demonstrated. It is anticipated that data compiled in this review will not only update researchers about the pharmacologic significance of carboxyxanthones, but also guide the design for the synthesis of new bioactive xanthone derivatives with improved medicinal properties.

## Figures and Tables

**Table 1:**
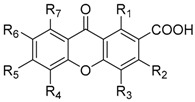
Structure of 9-oxo-9*H*-xanthene-2-carboxylic acid (**162**) and analogues (**163**–**284**).

Comp.	R_1_	R_2_	R_3_	R_4_	R_5_	R_6_	R_7_	REF
**162**	H	H	H	H	H	H	H	[151,152,153,154]
**163**	OMe	H	H	H	H	H	H	[155]
**164**	H	OMe	H	H	H	H	H	[155]
**165**	H	H	OMe	H	H	H	H	[155]
**166**	H	H	H	H	H	H	OMe	[155,169]
**167**	H	H	H	OMe	H	H	H	[155]
**168**	H	H	H	H	H	OMe	H	[155]
**169**	H	H	H	H	OMe	H	H	[155,163,167,169,172]
**170**	H	H	H	H	H	Me	H	[155]
**171**	H	H	H	H	H	C_2_H_5_	H	[155]
**172**	H	H	H	H	H	C_3_H_7_	H	[155]
**173**	H	H	H	H	H	*i*-C_3_H_7_	H	[155]
**174**	H	H	H	H	H	*sec*-C_4_H_9_	H	[155]
**175**	H	H	H	H	H	C_5_H_11_	H	[155]
**176**	H	H	H	*i*-C_3_H_7_	H	H	H	[155]
**177**	H	H	H	H	H	F	H	[155]
**178**	H	H	H	H	H	Cl	H	[153,155]
**179**	H	H	H	H	H	OH	H	[155]
**180**	H	H	H	H	H	OC_2_H_5_	H	[155]
**181**	H	H	H	H	H	OC_3_H_7_	H	[155]
**182**	H	H	H	H	H	*i*-OC_3_H_7_	H	[155]
**183**	H	H	H	H	H	OC_4_H_9_	H	[155]
**184**	H	H	H	*i*-OC_3_H_7_	H	H	H	[155]
**185**	H	H	H	H	H	COOH	H	[155,156]
**186**	H	H	H	H	H	OCH_2_CH(OH)CH_2_SPh	H	[157]
**187**	H	H	H	H	H	OCH_2_CH(OH)CH_2_S(4-F-Ph)	H	[157]
**188**	H	H	H	H	H	OCH_2_CH(OH)CH_2_S(4-Cl-Ph)	H	[157]
**189**	H	H	H	H	H	OCH_2_CH(OH)CH_2_S(3,4-Cl_2_-Ph)	H	[157]
**190**	H	H	H	H	H	OCH_2_CH(OH)CH_2_S(4-Br-Ph)	H	[157]
**191**	H	H	H	H	H	OCH_2_CH(OH)CH_2_S(4-OCH_3_-Ph)	H	[157]
**192**	H	H	H	H	H	OCH_2_CH(OH)CH_2_SCH_3_	H	[157]
**193**	H	H	H	H	H	OCH_2_CH(OH)CH_2_SC_2_H_4_OH	H	[157]
**194**	H	H	H	H	H	OCH_2_CH(OH)CH_2_SCH(CH_3_)_2_	H	[157]
**195**	H	H	H	H	H	OCH_2_CH(OH)CH_2_SC(CH_3_)_3_	H	[157]
**196**	H	H	H	H	H	OCH_2_CH(OH)CH_2_SC_6_H_11_	H	[157]
**197 ^a^**	H	H	H	H	H	OCH_2_CH(OH)CH_2_S(1-adm)	H	[157]
**198**	H	H	H	H	H	OCH_2_CH(OH)CH_2_SC_7_H_15_	H	[157]
**199**	H	H	H	H	H	OCH_2_CH(OH)CH_2_OH	H	[157,161]
**200**	H	H	H	H	H	OCH_2_CH(OH)CH_2_OCH_3_	H	[157]
**201**	H	H	H	H	H	OCH_2_CH(OH)CH_2_OC_2_H_4_OH	H	[157]
**202**	H	H	H	H	H	OCH_2_CH(OH)CH_2_OC_2_H_4_OCH_3_	H	[157]
**203**	H	H	H	H	H	OCH_2_CH(OH)CH_2_OCH_2_OF_3_	H	[157]
**204**	H	H	H	H	H	OCH_2_CH(OH)CH_2_SOC_6_H_5_	H	[157]
**205**	H	H	H	H	H	OCH_2_CH(OH)CH_2_SOCH_3_	H	[157]
**206**	H	H	H	H	H	COCH_3_	H	[158]
**207**	H	H	H	H	H	COC_2_H_5_	H	[158]
**208**	H	H	H	H	H	*i*-COC_3_H_7_	H	[158]
**209 ^b^**	H	H	H	H	H	COC_3_H_5_	H	[158]
**210 ^c^**	H	H	H	H	H	COC_5_H_9_	H	[158]
**211**	H	H	H	H	H	SH	H	[158]
**212**	H	H	H	SOCH_3_	H	H	H	[158]
**213**	H	H	H	*i*-SOC_3_H_7_	H	H	H	[158]
**214**	H	H	H	SCH_3_	H	H	H	[158]
**215**	H	H	H	*i*-SC_3_H_7_	H	H	H	[158]
**216**	H	H	H	SO_2_CH_3_	H	H	H	[158]
**217**	H	H	H	OMe	H	OMe	H	[158]
**218**	H	H	H	H	OMe	OMe	H	[158]
**219 ^c^**	H	H	H	H	OMe	H	OMe	[158]
**220**	H	H	H	Me	H	Me	H	[158]
**221**	H	H	H	H	Me	Me	H	[158]
**222**	H	H	H	H	H	Me	Me	[158]
**223**	H	H	H	OMe	H	SCH_3_	H	[158]
**224**	H	H	H	OEt	H	SOCH_3_	H	[158]
**225**	H	H	H	OC_3_H_7_	H	SOCH_3_	H	[158]
**226**	H	H	H	*i*-OC_3_H_7_	H	SOCH_3_	H	[158]
**227**	H	H	H	OC_4_H_9_	H	SOCH_3_	H	[158]
**228**	H	H	H	OC_5_H_11_	H	SOCH_3_	H	[158,159]
**229**	H	H	H	*i*-OC_5_H_11_	H	SOCH_3_	H	[158]
**230**	H	H	H	OC_5_H_9_	H	SOCH_3_	H	[158]
**231**	H	H	H	OC_8_H_17_	H	SOCH_3_	H	[158]
**232**	H	H	H	H	H	SCH_3_	H	[159]
**233**	H	H	H	C_6_H_13_	H	SCH_3_	H	[159,160]
**234**	H	H	H	OC_5_H_11_	H	SCH_3_	H	[159]
**235**	H	H	H	H	H	SOCH_3_	H	[159,161]
**236**	H	H	H	C_6_H_13_	H	SOCH_3_	H	[159]
**237**	H	H	H	H	H	SO(=NH)CH_3_	H	[159]
**238**	H	H	H	C_6_H_13_	H	SO(=NH)CH_3_	H	[159,160]
**239**	H	H	H	OC_5_H_11_	H	SO(=NH)CH_3_	H	[159]
**240**	H	H	H	H	H	SO(=NCONH_2_)CH_3_	H	[159]
**241**	H	H	H	C_6_H_13_	H	SO(=NCONH_2_)CH_3_	H	[159]
**242**	H	H	H	H	H	SO(=NCOPh)CH_3_	H	[159]
**243**	H	H	H	H	H	SO(=NCOCH_3_)CH_3_	H	[159]
**244**	H	H	H	H	H	SO(=NCOOC_2_H_5_)CH_3_	H	[159]
**245 ^d^**	H	H	H	H	H	SO(=N-Tos)CH_3_	H	[159]
**246**	H	H	H	H	H	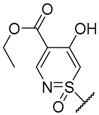	H	[159]
**247 ^d^**	H	H	H	H	H	S(=N-Tos)CH_3_	H	[159]
**248**	H	H	H	H	H	SO_2_Cl	H	[161]
**249**	H	H	H	H	H	SO_3_H	H	[161]
**250**	H	H	H	H	H	SO_2_NH_2_	H	[161]
**251**	H	H	H	H	H	SO_2_NHCH_3_	H	[161]
**252**	H	H	H	H	H	SO_2_NH(CH_3_)_2_	H	[161]
**253**	H	H	H	H	H	SO_2_NH(CH_3_)C_2_H_5_	H	[161]
**254**	H	H	H	H	H	SO_2_NH-*i*-C_3_H_8_	H	[161]
**255**	H	H	H	H	H	SO_2_NH(CH_3_)-*i*-C_3_H_8_	H	[161]
**256**	H	H	H	H	H	SO_2_NH(CH_3_)-*i*-C_4_H_9_	H	[161]
**257 ^e^**	H	H	H	H	H	SO_2_-pyrr	H	[161]
**258 ^f^**	H	H	H	H	H	SO_2_-morp	H	[161]
**259**	H	H	H	H	H	SO_2_NHC_2_H_4_OH	H	[161]
**260**	H	H	H	H	H	SO_2_NH(CH_3_)C_2_H_4_OH	H	[161]
**261**	H	H	H	H	H	SO_2_NH(C_2_H_4_OH)_2_	H	[161]
**262**	H	H	H	H	H	SC_2_H_4_OH	H	[161]
**263**	H	H	H	H	H	SOC_2_H_4_OH	H	[161]
**264**	H	H	H	H	H	SO_2_C_2_H_4_OH	H	[161]
**265**	H	H	H	H	H	SOC_2_H_4_OCH_3_	H	[161]
**266**	H	H	H	H	H	CH(OH)CH_3_	H	[161]
**267**	H	H	H	H	H	CH(OCH_3_)CH_3_	H	[161]
**268**	H	H	H	*i*-C_3_H_8_	H	*i*-C_3_H_8_	H	[162]
**269**	H	H	H	*t*-C_4_H_9_	H	*t*-C_4_H_9_	H	[162]
**270**	H	H	H	H	OC_10_H_21_	C_2_H_4_COOH	H	[163]
**271**	H	H	H	C_2_H_4_COOH	OC_10_H_21_	H	H	[163]
**272**	H	H	H	C_2_H_4_COOH	OC_4_H_8_CH=CH(4-OMe-Ph)	H	H	[163]
**273**	H	H	H	C_2_H_4_COOH	OC_3_H_6_O(4-COCH_3_-2-Et-5-OH-Ph)	H	H	[163,164]
**274**	H	H	H	C_2_H_4_COOH	OC_3_H_6_O(5-Et-4′-F-2-OH-1,1′-Ph_2_)	H	H	[164,165]
**275**	H	H	H	COOH	H	H	H	[154]
**276**	H	H	H	COOH	H	NO_2_	H	[154]
**277**	H	H	H	H	H	NO_2_	H	[154]
**278**	H	H	NO_2_	H	H	NO_2_	H	[154]
**279**	H	H	NO_2_	COOH	H	NO_2_	H	[154]
**280**	H	H	H	H	H	NH_2_	H	[154]
**281**	H	H	OCOCH_3_	H	H	H	H	[166]
**282**	H	H	OCOCH_3_	OCOCH_3_	H	H	H	[166]
**283**	H	H	OH	OH	H	H	H	[166]
**284**	H	H	NH_2_	NO_2_	H	*tert*-Butyl	H	[168]

^a^ adm—Adamantyl; ^b^ C_3_H_5_—Cyclopropyl; ^c^ C_5_H_9_—Cyclopentyl; ^d^ Tos—Tosyl; ^e^ pyrr—Pyrrolidino; ^f^ morp—Morpholino; Me—Methyl; Et—Ethyl; Ph—Phenyl.

**Table 2:**
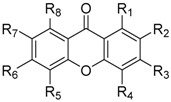
Structures of other 9-oxo-9*H*-xanthene carboxylic acid derivatives (**285**–**338**).

Comp.	R_1_	R_2_	R_3_	R_4_	R_5_	R_6_	R_7_	R_8_	REF
**285**	COOH	H	H	H	H	H	H	H	[151,154]
**286**	H	H	COOH	H	H	H	H	H	[151,154]
**287**	H	H	H	COOH	H	H	H	H	[151,154]
**288**	H	COC_2_H_4_COOH	H	H	H	H	H	H	[152]
**289**	H	H	OCH_2_COOH	H	H	H	H	H	[169,174]
**290**	H	H	OCH_2_COOH	H	H	H	H	F	[167,174]
**291**	H	H	OCH_2_COOH	Cl	H	H	H	F	[174]
**292**	H	H	OCH_2_COOH	H	H	H	H	F	[174]
**293**	H	H	OCH_2_COOH	Me	H	H	H	F	[174]
**294**	H	H	OCH_2_COOH	Cl	H	H	H	Cl	[174]
**295**	H	H	OCH_2_COOH	Cl	H	H	Cl	H	[174]
**296**	H	H	OCH_2_COOH	Cl	H	Cl	H	H	[174]
**297**	H	H	OCH_2_COOH	Cl	Cl	H	H	H	[174]
**298**	Cl	Cl	OCH_2_COOH	H	H	H	H	H	[174]
**299**	H	Cl	OCH_2_COOH	Cl	H	H	H	H	[174]
**300**	Cl	H	OCH_2_COOH	H	H	H	H	H	[174]
**301**	H	Cl	OCH_2_COOH	H	H	H	H	H	[174]
**302**	H	H	OCH_2_COOH	Cl	H	H	H	H	[174]
**303**	Me	H	OCH_2_COOH	H	H	H	H	H	[174]
**304**	H	Me	OCH_2_COOH	H	H	H	H	H	[174]
**305**	H	H	OCH_2_COOH	Me	H	H	H	H	[174]
**306**	H	Br	OCH_2_COOH	H	H	H	H	H	[174]
**307**	H	H	OCH(COOH)CH_2_		H	H	H	H	[174]
**308**	H	H	OCH(COOH)CH_2_		H	H	H	F	[174]
**309**	H	H	OCH(COOH)CH_2_		H	H	H	Cl	[174]
**310**	H	Cl	OCH(COOH)CH_2_		H	H	H	H	[174]
**311**	Cl	H	OCH(COOH)CH_2_		H	H	H	H	[174]
**312**	H	Me	OCH(COOH)CH_2_		H	H	H	H	[174]
**313**	Me	H	OCH(COOH)CH_2_		H	H	H	H	[174]
**314**	Br	H	OCH(COOH)CH_2_		H	H	H	H	[174]
**315**	Cl	Me	OCH(COOH)CH_2_		H	H	H	H	[174]
**316**	H	CH2CH(COOH)O		Cl	H	H	H	F	[174]
**317**	H	CH2CH(COOH)O		Me	H	H	H	F	[174]
**318**	H	CH2CH(COOH)O		Cl	H	H	H	Cl	[174]
**319**	H	CH2CH(COOH)O		Cl	H	H	H	H	[174]
**320**	H	CH2CH(COOH)O		Me	H	H	H	H	[174]
**321**	H	H	H	COOH	H	OC_10_H_21_	C_2_H_4_COOH	H	[163]
**322**	H	H	H	COOH	C_2_H_4_COOH	OC_10_H_21_	H	H	[163]
**323**	H	H	H	H	C_2_H_4_COOH	OC_3_H_6_O-(5-Et-4′-F-2-OH-1,1′-Ph_2_)	H	H	[164]
**324**	COOH	H	H	H	H	H	NO_2_	H	[154]
**325**	H	H	COOH	H	H	H	NO_2_	H	[154]
**326**	H	H	H	COOH	H	H	NO_2_	H	[154]
**327**	H	H	COOH	COOH	H	H	NO_2_	H	[154]
**328**	COOH	NO_2_	H	H	H	H	NO_2_	H	[154]
**329**	H	NO_2_	COOH	H	H	H	NO_2_	H	[154]
**330**	H	NO_2_	H	COOH	H	H	NO_2_	H	[154]
**331**	H	NO_2_	COOH	COOH	H	H	NO_2_	H	[154]
**332**	H	H	COOH	H	H	H	NH_2_	H	[154]
**333**	H	H	OC(CH_3_)_2_COOH	H	CH_3_	H	H	H	[175]
**334**	H	H	OCH_2_COOH	H	H	H	CH_3_	H	[175]
**335**	H	H	OCH_2_COOH	H	CH_3_	H	H	H	[175]
**336**	H	H	OCH(CH_3_)COOH	H	H	H	CH_3_	H	[175]
**337**	H	H	OC(CH_3_)_2_COOH	H	H	H	CH_3_	H	[175]
**338**	H	H	H	OCH(CH_3_)COOH	H	Cl	H	H	[175]
